# Prolonged Sleep Restriction Affects Glucose Metabolism in Healthy Young Men

**DOI:** 10.1155/2010/108641

**Published:** 2010-04-19

**Authors:** Wessel M. A. van Leeuwen, Christer Hublin, Mikael Sallinen, Mikko Härmä, Ari Hirvonen, Tarja Porkka-Heiskanen

**Affiliations:** ^1^Brain and Work Research Centre, Finnish Institute of Occupational Health, Topeliuksenkatu 41 a A, 00250 Helsinki, Finland; ^2^Department of Physiology, Institute of Biomedicine, University of Helsinki, PO Box 63, 00014 Helsinki, Finland; ^3^Centre of Expertise for Health and Work Ability, Finnish Institute of Occupational Health, Topeliuksenkatu 41 a A, 00250 Helsinki, Finland

## Abstract

This study identifies the effects of sleep restriction and subsequent recovery sleep on glucose homeostasis, serum leptin levels, and feelings of subjective satiety. Twenty-three healthy young men were allocated to a control group (CON) or an experimental (EXP) group. After two nights of 8 h in bed (baseline, BL), EXP spent 4 h in bed for five days (sleep restriction, SR), followed by two nights of 8 h (recovery, REC). CON spent 8 h in bed throughout the study. Blood samples were taken after the BL, SR, and REC period. In EXP, insulin and insulin-to-glucose ratio increased after SR. IGF-1 levels increased after REC. Leptin levels were elevated after both SR and REC; subjective satiety remained unaffected. No changes were observed in CON. The observed increase of serum IGF-1 and insulin-to-glucose ratio indicates that sleep restriction may result in an increased risk to develop type 2 diabetes.

## 1. Introduction

Sleep is considered to be a restorative process with beneficial effects on many bodily systems, including the digestive system, the immune system, and the cardiovascular system. Yet in modern industrialized societies, voluntary restriction of sleep is getting increasingly common due to, for instance, increasing work demands and atypical working hours [1]. Moreover, partial loss of sleep is common among people who experience environmental or psychological stress, who have psychiatric or physical disorders or who participate in shift work [2]. The consequences of this chronic deficiency of sleep are numerous and include increasing amounts of accidents, both in traffic and at work, increased prevalence of certain diseases, and even increased mortality [2]. It is important to understand and elucidate the mechanisms through which sleep and health are related if we are to find ways to manage people with chronically restricted sleep.

Sufficient sleep is a key component in the regulation of energy metabolism. Several epidemiological studies have shown that habitual short sleep duration is correlated with an increased risk of developing obesity and diabetes [3, 4]. Controlled laboratory studies, investigating the effects of prolonged sleep restriction on energy metabolism, are more scarce. Glucose tolerance has been shown to be impaired after six days of sleep restricted to four hours per night, compared to a condition in which participants were allowed twelve hours in bed per night for six days [5], which might contribute to the risk of developing type 2 diabetes. Furthermore, it has been shown that two nights of sleep restricted to four hours, compared to two nights of ten hours in bed, results in a reduction of the satiety hormone leptin, accompanied by increased hunger and increased serum concentrations of the orexigenic factor ghrelin [6], which might add to the risk of developing obesity. 

Rodent studies on energy metabolism have mainly applied either a total sleep deprivation or a selective REM sleep deprivation design, which are both difficult to compare to a sleep restriction design. Everson and Crowley showed, in rats, that 15 days of sleep restriction suppress concentrations of IGF-1 and leptin [7], which was, interestingly, accompanied by weight loss. Bodosi and colleagues have shown, on the other hand, that 5 h sleep deprivation does not affect leptin concentrations but increases ghrelin concentrations [8].

In the present study, we simulated accumulating sleep restriction during five working days followed by two days of weekend recovery sleep and measured the changes in several metabolic parameters that occurred during this period, including glucose metabolism, serum leptin concentrations, and feelings of satiety.

## 2. Materials and Methods

### 2.1. Participants

Twenty-three healthy men, aged 19–29 (mean ± SD 23.1 ± 2.5), participated in this study and were recruited by advertisements in local newspapers during a two-year time span. First, volunteers were screened during a telephone interview, followed by a thorough physical examination, blood tests (triglycerides, cholesterol, haemoglobin, creatinine, leukocytes, erythrocytes, haematocrit, TSH, ASAT, ALAT, MCV, MCH, and MCHC), and screening polysomnography. Participants' final eligibility was evaluated according to preset inclusion and exclusion criteria. Volunteers were excluded from participation for any of the following: an irregular sleep-wake schedule, regular naps, having either advanced or delayed sleep phase syndrome, insomnia or other sleep problems, loud snoring >5 nights/week, repeating apneas, excessive daytime sleepiness (Epworth Sleepiness Scale >8), restless legs at least once a month, a disorder that might become worse because of prolonged wakefulness (such as a severe mental disorder, epilepsy, and cardiac arrhythmia), excessive caffeine consumption (>5 cups of coffee/day), excessive alcohol consumption (>15 units/week; 1 unit = 11 g or 13.9 mL of alcohol), smoking, medication affecting the central nervous system during the last two weeks, any clinically relevant abnormality on blood tests, any other reason that health may be harmed because of if participating, apnea-hypopnea index >20, periodic limb movement index >25, epileptiform activity on the EEG, abnormal urinary drug screening, and having experienced a significant recent life event that could disturb sleep. In addition, volunteers were only included when fulfilling all of the following criteria: male aged 19–29, sleep latency in the evening <20–30 minutes, uninterrupted nocturnal sleep and if awakened no problem to fall asleep again, no chronic disease or symptom affecting sleep, no continuous medication, and willing and able to participate. 

All participants reported habitual sleep duration of 7–9 hours and a regular sleep-wake schedule. For at least one week prior to the experiment they completed sleep diaries and carried actigraphs in order to verify adherence to a regular sleep-wake schedule. One week prior to the start of the experiment, participants had an adaptation night in the sleep laboratory. The prestudy mean (±SD) sleep duration was 6.88 (±0.58) h in the control group and 7.05 (±0.80) h in the experimental group. Participant's prestudy mean (±SD) body mass index (BMI) was 23.24 (±2.39) in the control group and 23.25 (±2.70) in the experimental group.

### 2.2. Experimental Protocol

**Figure 1:**
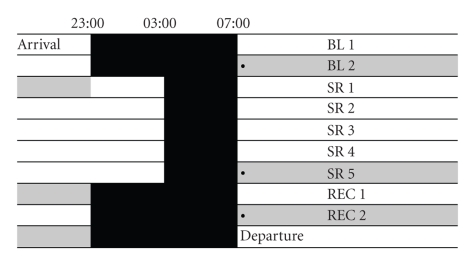
The experimental protocol. After two nights of 8 h sleep (baseline BL), sleep is restricted to 4 h per night for 5 subsequent days (sleep restriction, SR), followed by three nights of 8 h recovery sleep (REC). Profile days are shaded in grey. Bullet points indicate the taking of blood samples and rating of subjective satiety.

The protocol was approved by the ethical committee of the University Hospital of Helsinki District, and written confirmed consent was obtained from all participants. A 10-day experimental schedule (Figure 1) was executed at the Brain and Work Research Centre of the Finnish Institute of Occupational Health (FIOH). Altogether, participants spent ten consecutive nights in the sleep laboratory. Fifteen participants were randomly allocated to the experimental group (EXP), spent the first two nights 8 h in bed (baseline, BL; from 23:00 h to 07:00 h), followed by five nights of 4 h in bed (sleep restriction, SR; from 03:00 h to 07:00 h) and, finally, again three nights of 8 h in bed (recovery, REC). Eight participants were randomly allocated to the control group (CON) and spent 8 h in bed every night. Sleep during the daytime was not allowed, which was monitored by EEG recordings and a continuously present investigator. During waking, participants took part in a bigger experiment of our sleep laboratory involving the simulation of a workweek by a variety of cognitive and psychological tasks. Their main activities during the day included the repeated assessment of the psychomotor vigilance task (PVT), the Brain@Work Multitask, a saccade test, and the training and testing of memory and motor tasks. Moreover, saliva samples were provided ten times per day and blood pressure was assessed eight times a day.

Participants ate standardized meals at fixed times throughout the experiment: breakfast at 08:00 h (600 kcal), lunch at 12:30 h (800 kcal), dinner at 18:30 h (700 kcal); snacks at 15:30 h (300 kcal) and 21:30 h (200 kcal). In addition, participants in EXP ate a piece of fruit (apple or orange) at 00:30 h (50 kcal). Participants were not allowed to leave the building but could, during regular short breaks, leave the sleep and test room and visit a relax room with television and PC. Illuminance in the sleep and test room ranged from 150 to 400 lux, and in the relax room from 350 to 600 lux, the temperature in the rooms ranged from 19 to 23 degrees Celsius. Polygraphy and ECG were continuously measured.

### 2.3. Hormonal Measurements

Hormonal levels were assessed from blood samples that were taken before breakfast at 07:30 h after the second BL night, the fifth SR night, and the second REC night in EXP and corresponding nights in CON. Samples were analyzed by Medix Laboratories, Espoo, Finland for glucose, insulin, IGF1, and leptin. Before blood sampling, subjects were asked to rate their feeling of hungriness on a 1 to 5 scale (1 = very hungry, 5 = very satiated). The saliva samples described above were analyzed for cortisol levels using a commercial kit assay (Salivary Cortisol, LIA, IBL, Hamburg, Germany).

### 2.4. Statistical Analysis

For both CON and EXP, mean values ± SD were calculated for each experimental day, BL, SR, and REC. In addition, SR and REC values were expressed as percentages of each individual participant's BL value, that is, normalized. We have compared SR and REC values to BL values by applying paired *t*-tests for normally distributed differences and Wilcoxon signed ranks tests for differences that were not normally distributed. The normality of differences was checked using Kolmogorov-Smirnov goodness of fit test. A *P*-value <.05 was considered to be statistically significant. All statistical analyses were carried out using SPSS version 15 (SPSS Inc., Chicago, USA).

## 3. Results

### 3.1. Total Sleep Duration and Cortisol Profile

In CON, the mean total sleep duration (±SD) remained unaffected throughout the experiment, whereas in EXP, the mean sleep duration, as expected, strongly reduced during the SR period (Table 1). In EXP, the peak in cortisol levels was delayed with 16.2 ± 5.5 min. after SR compared to BL (*P* < .05; Table 1). After REC, the cortisol profile was similar to BL again. In CON, the cortisol profile remained unaffected throughout the experiment (Table 1).

### 3.2. Glucose, Insulin, and IGF-1

**Figure 2:**
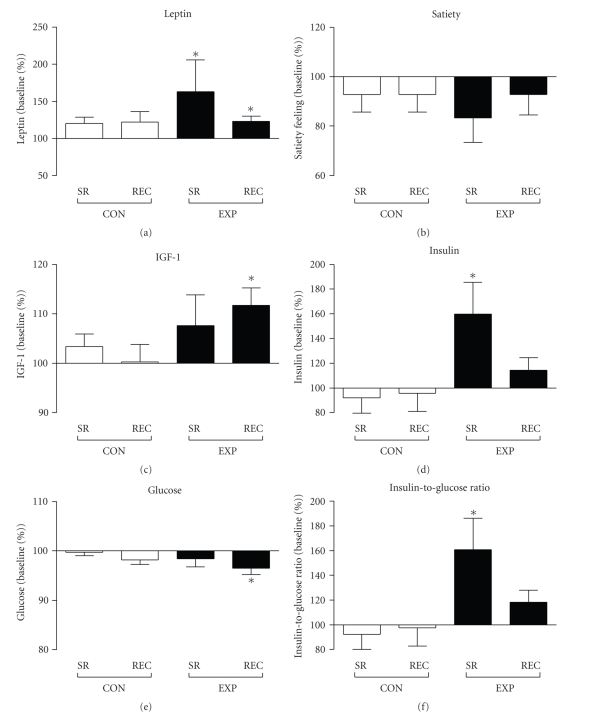
Changes in serum concentrations of leptin, IGF-1, insulin, and glucose, and changes in insulin-to-glucose ratio and subjective satiety after sleep restriction (SR) and recovery (REC) in the control group (CON) and experimental group (EXP). Data are expressed as percentages of participant's individual baseline values (mean ± SEM) (*P < .05).

Mean glucose, insulin, and IGF-1 levels throughout the experiment in both groups are described in Table 2. Glucose levels showed a tendency for a decrease after SR and its levels were significantly decreased after REC to 65.5% ± 1.3% of BL levels (*P* < .05; Figure 2). In CON, glucose levels remained at BL level throughout the experiment (Figure 2).

Insulin levels were increased after SR to 159.9% ± 25.6% of BL levels (*P* < .05; Figure 2) and returned back to BL levels after recovery (114.5% ± 10.1% of BL levels). In CON, insulin levels remained at BL level throughout the experiment (Figure 2).

The insulin-to-glucose ratio was significantly increased after SR to 160.8% ± 25.4% of BL levels (*P* < .05; Figure 2), returning back to BL levels after subsequent REC (118.2% ± 9.9% of BL levels). In CON, the insulin-to-glucose ratio remained at BL level throughout the experiment (Figure 2).

IGF-1 levels showed a tendency for an increase after SR and its levels were significantly elevated after REC to 111.7% ± 3.6% of BL levels (*P* < .01). In CON, IGF-1 levels remained at BL level throughout the experiment (Figure 2).

### 3.3. Leptin and Subjective Satiety

Mean leptin levels and feelings of subjective satiety throughout the experiment in both groups are described in Table 2. Leptin levels were increased after SR to 163.3% ± 42.4% of BL levels (*P* < .01) and were still significantly elevated after REC (123.1% ± 7.0% of BL levels; *P* < .01; Figure 2). In CON, leptin levels remained at BL level throughout the experiment (Figure 2).

Feelings of satiety remained unaffected throughout the experiment in both groups (Figure 2).

## 4. Discussion

Chronic sleep deprivation is becoming an increasingly common phenomenon in modern 24 h societies due to, for instance, voluntary sleep restriction and increasing work demands [1, 9]. Restricted sleep does not only result in sleepiness and impaired cognitive performance, it also adversely affects general health [10]. Several widespread disorders have been shown to be epidemiologically associated with habitual short sleep duration, including cardiovascular diseases [11, 12], type 2 diabetes [13], and obesity [14, 15].

In the present study, serum glucose levels declined during the course of sleep restriction and subsequent recovery sleep, whereas serum insulin levels increased. Hence, the insulin-to-glucose ratio was significantly elevated after sleep restriction but returned to baseline values after subsequent recovery sleep. Elevating insulin levels that are not accompanied by elevations in glucose levels indicate a reduced sensitivity to insulin, which may ultimately increase the risk of developing noninsulin-dependent diabetes (i.e., type 2 diabetes). Taken together with a previous study showing that prolonged sleep restriction significantly lowers glucose tolerance [5], the experimental support for a causative connection between insufficient sleep and type 2 diabetes is gradually accumulating and supports the already present epidemiological evidence [13, 16, 17].

Under normal physiological conditions, blood glucose concentrations are tightly regulated within narrow limits. A well-known condition in which blood glucose levels rise due to deficits in insulin signaling is diabetes, but no common conditions are known in which blood glucose levels decline. Blood glucose is the most important energy supply to the brain and, therefore, the observed decrease in glucose after recovery as compared to baseline is as puzzling as it is alarming, since the most important adverse effect of chronically decreased blood glucose levels is brain dysfunction and in extreme cases even damage [18]. Moreover, low levels of fasting blood glucose are associated with an increased mortality risk [19].

In addition to insulin, insulin-like growth factor-1 (IGF-1) is another substance that lowers serum levels of glucose in both rats and humans [20, 21] and has even the capability of doing so in patients with severe insulin resistance [22]. The present study has indeed shown that, after recovery, IGF-1 levels were increased while glucose levels were decreased. Interestingly, in addition to lowering serum glucose levels, IGF-1 has also been shown to decrease serum insulin levels in both rats and humans [23, 24]. It has been hypothesized that, by lowering insulin levels, IGF-1 reduces insulin resistance and might thus be of therapeutical importance in physiological states that are associated with insulin resistance, such as type 2 diabetes. Hence, the observed elevations in IGF-1 after recovery in the present study might be viewed as a compensatory reaction to the increased insulin levels after sleep restriction.

The rapidly expanding global incidence of obesity has a great impact on public health [25], for instance, by increasing the risk of developing cardiovascular diseases. Not only has this trend been paralleled by a trend of a gradual reduction in self-reported sleep duration, many epidemiological studies have linked those trends and observed a correlation between short sleep and obesity [26]. Recently, however, several groups have questioned the clinical relevance of this link [27, 28]. Expanding the current literature with experimental investigations might attribute to resolving those heated debates, editorials, and news reports.

Leptin and ghrelin are peripheral hormones believed to contribute to the central regulation of food intake [29]. Ghrelin, predominantly released by the stomach, stimulates appetite whereas leptin, mainly produced by adipocytes, stimulates feelings of satiety. Therefore, chronic elevations of ghrelin levels and/or reductions of leptin levels may attribute to the development of obesity. Obesity is indeed associated with leptin resistance and obese subjects show highly elevated serum concentrations of leptin [30]. Hitherto, only two experimental studies have investigated the effects of prolonged sleep restriction on serum ghrelin and leptin levels and observed decreased leptin and increased ghrelin levels, accompanied by increased feelings of hunger and appetite after a period of 4 h sleep compared to a period of 10 h sleep [6] and 12 h sleep [31]. In addition, prolonged sleep restriction in rats has been shown to result in decreased leptin levels that were associated with a reduction in body weight despite an increase in food intake [7]. Our observations, interestingly, contradict those findings in not having found any changes in feelings of hunger and having found an increase rather than a decrease in serum leptin levels.

Several factors are known to regulate serum leptin concentrations. Taheri and colleagues have shown that sleep duration is correlated to leptin levels [32]. Hence, long-sleepers have higher serum leptin concentrations than short-sleepers. We are, however, the first to show in a within-subject design that experimental restriction of participant's habitual sleep duration does not have a similar effect and that it is even increasing serum leptin concentrations. The only previous experimental studies compared sleep restriction against sleep extension and have found that after sleep restriction, leptin levels are lower than after sleep extension but unaffected as compared to participant's habitual sleep duration [6, 31].

Serum leptin concentrations are known to exhibit a circadian rhythm, with minimum values during daytime and a nocturnal rise [33]. This rhythm is not entrained to the circadian clock, but to meal patterns [34]. However, it does not acutely change in response to single meals [35]: a substantial meal of 1000 kcal did not alter leptin levels for the next three hours after administration [36]. In our study, there was only a modest (16 minute) delay in the endogenous circadian rhythm of salivary cortisol in the experimental group. However, meal timing was kept constant throughout the experiment, except for an additional apple or orange that participants in the experimental group received at 00:30 h during the days of restricted sleep. We find it unlikely that this small addition of about 50 kcal for a period of five days to the habitual meal pattern would have increased leptin levels with 63%. How abolishing oral meals completely by replacing them with intravenous glucose infusion affects serum leptin concentrations [6] is not known.

Physical activity is inversely related to fasting plasma leptin levels [37]. Physical exercise has indeed been shown to result in decreased concentrations of serum leptin, both acutely and over the entire 24 h time span [38, 39]. In the present study, however, we did not aim to keep physical activity constant. Hence, participants in the experimental group were not restricted in their physical activity during the period of prolonged wakefulness. Therefore, physical activity was slightly elevated during this period when compared to the baseline and recovery periods. This could, in theory, have decreased leptin levels.

Leptin has for a long time been considered to be purely a satiety signal and previous sleep restriction studies have indeed found that lower leptin levels were associated with increased feelings of hunger [6]. In the present study, however, elevated leptin levels were not accompanied by any changes in hunger feelings. This may suggest that leptin plays additional physiological roles apart from regulating food intake, such as a proinflammatory role [40, 41].

An alternative explanation for the observed epidemiological correlation between short sleep and obesity might have little to do with the homeostatic control of sleeping and feeding behavior. As Saper and colleagues have pointed out, both the regulation of feeding and sleeping have a strong hedonic component [42, 43]. That is, both can be very satisfying at times when their physiological need is not that strong. It may be that, under the unpleasant experience of restricted sleep, a search for pleasure begins and excessive food is being consumed. Indeed, it has been shown that sleep restricted subjects—in a setting of ad libitum access to palatable food—consume excessive amounts of calories from snacks [44].

## 5. Conclusions

We showed that prolonged sleep restriction in a situation that mimics a working week changes glucose metabolism and may lead to an increased risk of developing type 2 diabetes. Two nights of normal sleep, however, restored this effect. In addition, we showed that five nights of sleep restriction does not affect hunger feelings and results in elevated leptin levels. This suggests that sleep restriction per se as it would occur during a typical working week may not increase the risk of developing obesity. Therefore, the previously observed epidemiological associations between short sleep and obesity might be due to a common underlying factor rather than a direct causation between short sleep and obesity. In addition, the excessive consumption of calories from snacks rather than from meals during a period of restricted sleep may contribute to the development of weight gain and/or obesity [44].

## Figures and Tables

**Table 1:** Sleep duration and cortisol.

Variable	Group	Day		
		BL	SR	REC
		Mean (±SD)	Mean (±SD)	Mean (±SD)
Total sleep duration (min.)	CON	440 (±23)	442 (±16)	429 (±27)
	EXP	439 (±20)	232 (±5)	458 (±13)

Cortisol peak (clock time)	CON	07:48 (±0:15)	07:33 (±0:19)	07:33 (±0:10)
	EXP	07:39 (±0:14)	07:55 (±0:11)	07:36 (±0:13)

**Table 2:** Descriptives of the data.

Variable	Group	Day		
		BL	SR	REC
		Mean (±SD)	Mean (±SD)	Mean (±SD)
Glucose (mmol/L)	CON	5.14 (±0.50)	5.13 (±0.49)	5.04 (±0.41)
	EXP	4.91 (±0.24)	4.83 (±0.24)	4.74 (±0.17)

Insulin (mU/L)	CON	8.27 (±2.62)	7.33 (±2.84)	7.50 (±2.82)
	EXP	6.02 (±2.90)	8.59 (±6.99)	6.25 (±2.36)

Insulin-to-glucose ratio	CON	1.65 (±0.65)	1.49 (±0.73)	1.52 (±0.67)
	EXP	1.22 (±0.57)	1.75 (±1.33)	1.31 (±0.46)

IGF-1 (nmol/L)	CON	33.81 (±11.15)	34.84 (±10.99)	34.03 (±11.63)
	EXP	26.35 (±6.29)	27.64 (±5.39)	28.93 (±5.23)

Leptin (*μ*g/L)	CON	3.44 (±3.09)	3.79 (±2.92)	3.70 (±2.80)
	EXP	6.25 (±4.55)	7.59 (±5.00)	6.93 (±4.07)

Satiety (1 to 5 scale)	CON	2.71 (±0.76)	2.57 (±0.98)	2.57 (±0.98)
	EXP	2.21 (±0.43)	1.79 (±0.70)	2.00 (±0.55)
